# Differentiating primary central nervous system lymphoma from glioblastoma by time-dependent diffusion using oscillating gradient

**DOI:** 10.1186/s40644-023-00639-7

**Published:** 2023-11-30

**Authors:** Kiyohisa Kamimura, Tsubasa Nakano, Tomohito Hasegawa, Masanori Nakajo, Chihiro Yamada, Yoshiki Kamimura, Kentaro Akune, Fumitaka Ejima, Takuro Ayukawa, Hiroaki Nagano, Koji Takumi, Masatoyo Nakajo, Nayuta Higa, Hajime Yonezawa, Ryosuke Hanaya, Mari Kirishima, Akihide Tanimoto, Takashi Iwanaga, Hiroshi Imai, Thorsten Feiweier, Takashi Yoshiura

**Affiliations:** 1 Department of Advanced Radiological Imaging, Kagoshima University Graduate School of Medical and Dental Sciences, 8-35-1 Sakuragaoka, Kagoshima, 890-8544 Japan; 2 Department of Radiology, Kagoshima University Graduate School of Medical and Dental Sciences, 8-35-1 Sakuragaoka, Kagoshima, 890-8544 Japan; 3 Department of Neurosurgery, Kagoshima University Graduate School of Medical and Dental Sciences, 8-35-1 Sakuragaoka, Kagoshima, 890-8544 Japan; 4 Department of Pathology, Kagoshima University Graduate School of Medical and Dental Sciences, 8-35-1 Sakuragaoka, Kagoshima, 890-8544 Japan; 5 Department of Radiological Technology, Kagoshima University Hospital, 8-35-1 Sakuragaoka, Kagoshima, 890-8544 Japan; 6 Siemens Healthcare K.K., Gate City Osaki West Tower, 1-11-1 Osaki, Shinagawa-Ku, Tokyo, 141-8644 Japan; 7 Siemens Healthcare GmbH, Henkestrasse 127, 91052 Erlangen, Germany

**Keywords:** Diffusion, Glioblastoma, Magnetic resonance imaging, Primary central nervous system lymphoma

## Abstract

**Background:**

This study aimed to elucidate the impact of effective diffusion time setting on apparent diffusion coefficient (ADC)-based differentiation between primary central nervous system lymphomas (PCNSLs) and glioblastomas (GBMs) and to investigate the usage of time-dependent diffusion magnetic resonance imaging (MRI) parameters.

**Methods:**

A retrospective study was conducted involving 21 patients with PCNSLs and 66 patients with GBMs using diffusion weighted imaging (DWI) sequences with oscillating gradient spin-echo (Δ_eff_ = 7.1 ms) and conventional pulsed gradient (Δ_eff_ = 44.5 ms). In addition to ADC maps at the two diffusion times (ADC_7.1 ms_ and ADC_44.5 ms_), we generated maps of the ADC changes (cADC) and the relative ADC changes (rcADC) between the two diffusion times. Regions of interest were placed on enhancing regions and non-enhancing peritumoral regions. The mean and the fifth and 95^th^ percentile values of each parameter were compared between PCNSLs and GBMs. The area under the receiver operating characteristic curve (AUC) values were used to compare the discriminating performances among the indices.

**Results:**

In enhancing regions, the mean and fifth and 95^th^ percentile values of ADC_44.5 ms_ and ADC_7.1 ms_ in PCNSLs were significantly lower than those in GBMs (*p* = 0.02 for 95^th^ percentile of ADC_44.5 ms_, *p* = 0.04 for ADC_7.1 ms_, and *p* < 0.01 for others). Furthermore, the mean and fifth and 95^th^ percentile values of cADC and rcADC were significantly higher in PCNSLs than in GBMs (each *p* < 0.01). The AUC of the best-performing index for ADC_7.1 ms_ was significantly lower than that for ADC_44.5 ms_ (*p* < 0.001). The mean rcADC showed the highest discriminating performance (AUC = 0.920) among all indices. In peritumoral regions, no significant difference in any of the three indices of ADC_44.5 ms_, ADC_7.1 ms_, cADC, and rcADC was observed between PCNSLs and GBMs.

**Conclusions:**

Effective diffusion time setting can have a crucial impact on the performance of ADC in differentiating between PCNSLs and GBMs. The time-dependent diffusion MRI parameters may be useful in the differentiation of these lesions.

## Background

Glioblastoma (GBM) is the most common and aggressive primary malignant brain tumor in adults [1]. In most cases with clinically and radiographically suspected GBMs, gross surgical resection is attempted. The incidence of primary central nervous system lymphomas (PCNSLs) has significantly increased in both immunosuppressed and immunocompetent individuals, although these lesions are less common than gliomas [2–4]. Unlike GBMs, PCNSLs are usually treated with chemotherapy and whole-brain radiotherapy, without extended surgical mass reduction. Therefore, pretreatment differentiation between PCNSLs and GBMs is essential for therapeutic decision-making. On magnetic resonance imaging (MRI), both GBMs and PCNSLs are typically observed as strongly enhanced masses, often accompanied by edema of the surrounding tissue [5–7]. Thus, their differentiation may be difficult. Diffusion weighted imaging (DWI) findings and apparent diffusion coefficient (ADC) measurements give valuable information regarding the microstructural organization. The ADC values are known to be inversely correlated with tumor cellularity [8]. Studies have shown that ADC values help differentiate between PCNSLs and GBMs [9–11]. PCNSL was characterized by lower ADC than GBM, presumably reflecting higher cellularity [12, 13].

Diffusion time is a basic parameter of DWI and represents the observation time of diffusion. In biological tissues, there are spatial barriers such as fibers and cell membranes that restrict the water molecular motions (restricted diffusion). Under the condition of the restricted diffusion, the ADC values increase when diffusion time decrease [14–17], as the molecules have less chance to collide with the barriers during the diffusion time. The conventional pulsed gradient spin-echo (PGSE) DWI sequences require a long diffusion time to achieve high b-values due to limited maximum gradient strength [18, 19]. Furthermore, the 180 pulse takes several milliseconds, so there is a limit to the minimum diffusion time in PGSE even if the gradient strength is unlimited. Thus, investigating the effects of diffusion time on ADC using a clinical MRI scanner has been difficult. The oscillating gradient spin-echo (OGSE) DWI sequence is an emerging diffusion encoding method [14], in which quickly oscillating gradients are used instead of the long diffusion-sensitizing gradients used in the PGSE method, thereby allowing for shorter diffusion times. Studies have investigated the diffusion time dependence of ADC using the PGSE and OGSE methods in combination, an approach called time-dependent diffusion MRI. Time-dependent diffusion MRI may add specific information regarding restricted diffusion in the tissue microstructure. To the best of our knowledge, the effect of the diffusion time on the diagnostic performance of ADC in differentiating between PCNSLs and GBMs has not been reported. Moreover, usefulness of the diffusion time dependence of ADC derived from the time dependent MRI for this purpose has never been investigated. Therefore, this study aimed to elucidate the impact of diffusion time setting on ADC-based differentiation between PCNSLs and GBMs and to determine whether time-dependent diffusion MRI is useful in the differentiation of these lesions.

## Materials and methods

### Patients

We retrospectively evaluated patients with PCNSLs or GBMs who underwent MRI examination between January 2019 and December 2022 at our institution. This retrospective study (approval no. 220126) was approved by our Institutional Review Board, and the need for written informed consent was waived because of the retrospective nature of this study.

Consecutive pathologically proven PCNSLs or GBMs based on the 2021 World Health Organization classification [20] were included in this study. All GBMs were diagnosed based on an integrated diagnosis, combining histology and a glioma-tailored next-generation sequencing panel developed by our institution [21]. The exclusion criteria were as follows: (a) lack of preoperative MRI, including DWI with both OGSE and PGSE sequences; (b) poor image quality; (c) masses smaller than 1 cm; (d) previous surgical resection or irradiation; or (e) lack of contrast-enhancing lesions.

In patients with multiple lesions, the largest mass was examined by MRI.

**Fig. 1:**
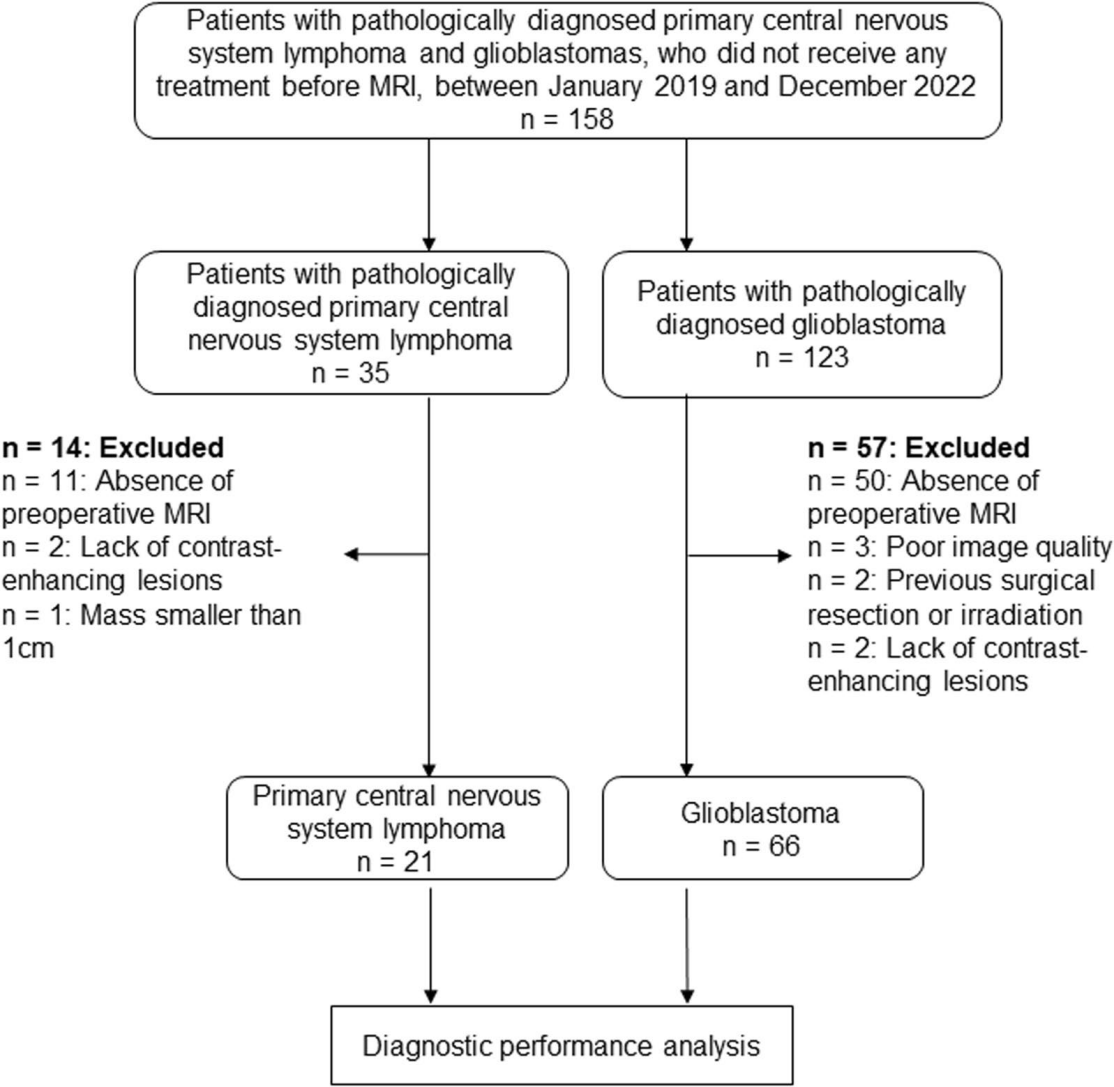
The diagram indicating the inclusion and exclusion criteria and the flow of the inclusion of eligible patients in this study

In this study, 158 consecutive patients (35 with PCNSLs and 123 with GBMs) were considered for inclusion. Seventy-one patients were excluded because of the absence of preoperative MRI, including both OGSE and PGSE DWI scans (11 with PCNSLs and 50 with GBMs), mass smaller than 1 cm (one with PCNSL), poor image quality caused by artifacts in the DWIs (three with GBMs), previous surgical resection or irradiation (two with GBMs), or the lack of contrast-enhancing lesions (two with PCNSLs and two with GBMs). We finally analyzed 21 patients with PCNSLs (15 men and six women; age range 40–87 years; mean age, 70 ± 13 years; 19 with primary diffuse large B-cell lymphoma of the central nervous system, and two with T-cell lymphomas) and 66 patients with isocitrate dehydrogenase-wildtype GMBs (36 men and 30 women; age range 15–92 years; mean age, 70 ± 13 years) (Fig. 1). All patients were diagnosed histopathologically after total or partial surgical resection. All patients were biopsy naive and had not received any treatment before MRI examination. No significant difference in the mean age (*p* = 0.71) or sex distribution (*p* = 0.21) was observed between patients with PCNSLs and those with GBMs.

In a previous study, we compared glioblastoma and metastatic brain tumors using the time-dependent diffusion MRI [22]. Patients with glioblastoma analyzed in the present study include 65 patients with glioblastoma used in our previous study.

### MRI acquisition

**Fig. 2:**
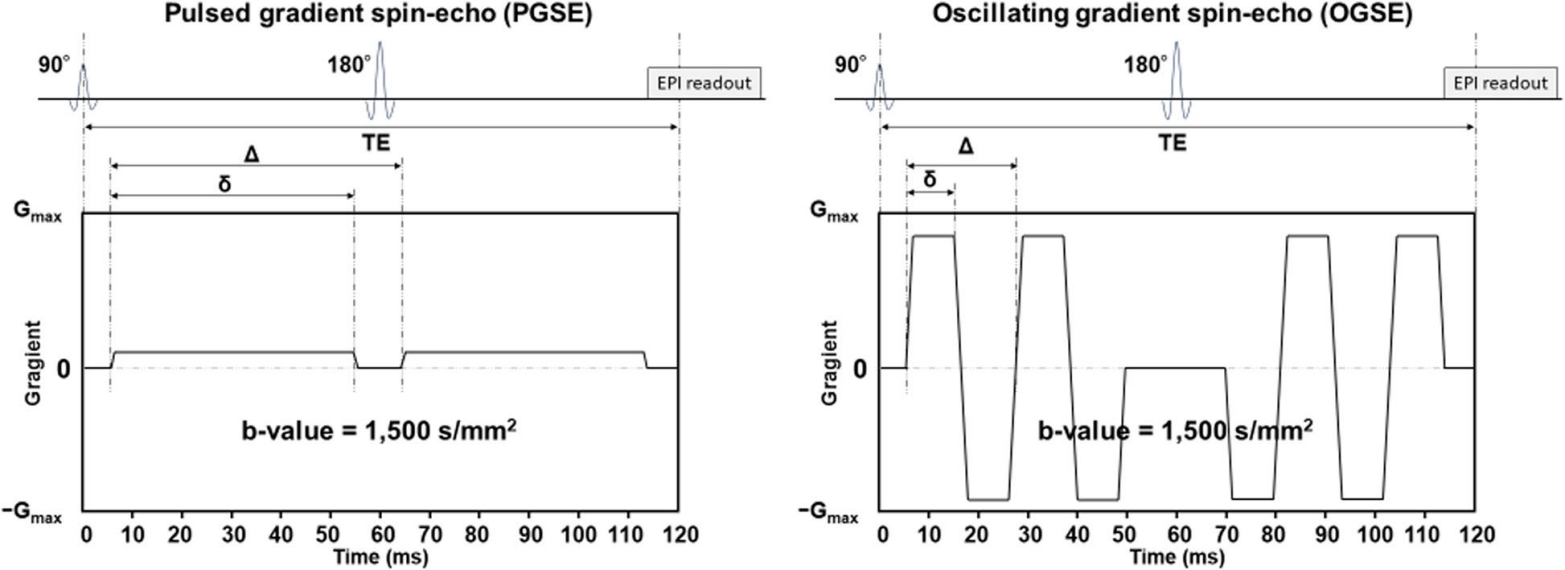
Schematic representation of the diffusion gradient waveforms for pulsed gradient spin-echo (PGSE) (left) and oscillating gradient spin-echo (OGSE) (right). G = gradient vector; Δ, diffusion gradient separation; δ, diffusion gradient pulse duration; EPI, echo planar imaging; TE, echo time

All patients were examined on a 3 T MR scanner (MAGNETOM Prisma; Siemens Healthcare; maximum gradient amplitude = 80 mT/m, maximum slew rate = 200 T/m/s for each gradient axis with a 20-channel head radiofrequency receive coil). DWI was scanned with research sequences for the OGSE DWI using b-values of 0 and 1,500 s/mm^2^ (number of repeated scans: 1 and 4, respectively) and three diffusion encoding directions. OGSE diffusion encoding used trapezoid-sine waveforms [23]. An effective diffusion time (Δ_eff_) of 7.1 ms (frequency = 50 Hz; diffusion gradient pulse duration [δ] = 8.5 ms) was used. PGSE DWI was also performed with b-values of 0 and 1,500 s/mm^2^ (number of repeated scans: 1 and 4, respectively) and three diffusion encoding directions. The Δ_eff_ for the PGSE encoding was 44.5 ms (diffusion gradient separation [Δ] = 59.8 ms; δ = 46.1 ms). The two sequences used the same parameters, as follows: repetition time (TR), 4,600 ms; echo time (TE), 120 ms; field of view (FOV), 230 × 230 mm^2^; matrix size, 72 × 72; and slice thickness, 5 mm. The acquisition times were 1 min and 13 s for PGSE DWI and 1 min and 19 s for OGSE DWI. Figure 2 shows the pulse sequence diagrams for PGSE and OGSE.

Post-contrast 2D T1-weighted spin-echo images were obtained using the following parameters: TR, 410 ms; TE, 10 ms; number of excitations, 1; matrix, 304 × 304 (reconstructed to 512 × 512); number of slices, 24; slice thickness, 5 mm; interslice gap, 1 mm; FOV, 230 × 230 mm^2^; and scan time, 2 min and 46 s. These images were used as the anatomical reference in delineating the region of interests (ROIs). Our routine MRI for central nervous system lesions included the following pre-contrast sequences (Table 1): 2D T1-weighted spin-echo imaging, 2D T2-weighted turbo spin-echo imaging, 2D fluid-attenuated inversion recovery (FLAIR) imaging, and 3D susceptibility-weighted imaging. Pre-contrast T1-weighted images were used to confirm contrast enhancement.

**Table 1:** Imaging parameters of pre- and post-contrast conventional MRI sequences

	Precontrast 2D T1-weighted imaging	2D T2-weighted imaging	2D fluid-attenuated inversion recovery imaging	3D susceptibility-weighted imaging	Postcontrast 2D T1-weighted imaging
Sequence	2D SE	2D TSE	2D IR-TSE	3D FLASH	2D SE
TR (ms)	520	4000	9000	28	520
TE (ms)	12	91	121	20	12
TI (ms)	N/A	N/A	2530	N/A	N/A
FA (degree)	70/180	150	120	15	70/180
Bandwidth (Hz/pixel)	181	199	130	120	181
Number of excitations	1	1	1	1	1
Turbo factor	N/A	9	25	N/A	N/A
Acceleration factor	N/A	2	2	2	N/A
FOV (mm)	230	230	230	230	230
Matrix	269 × 384	380 × 448	307 × 384	240 × 320	269 × 384
Thickness (mm)	5	5	5	2.5	5
Intersection gap (mm)	1	1	1	N/A	1
Acquisition time (s)	148	80	126	174	148

### Generating diffusion parametric maps

ADC values were calculated assuming the mono-exponential signal decay between lower and higher b-values.

According to previous studies [24, 25], we evaluated the ADC change (cADC) and the relative ADC change (rcADC) between OGSE (short diffusion time) and PGSE (long diffusion time). cADC and rcADC maps were generated via pixel-by-pixel calculation using the following formulas:

$$\begin{array}{l}\mathrm{cADC}={\mathrm{ADC}}_{7.1\mathrm{ms}}- {\mathrm{ADC}}_{44.5\mathrm{ms}}\\ \begin{array}{cc}\mathrm{rcADC}=\frac{\left({\mathrm{ADC}}_{7.1\mathrm{ms}}- {\mathrm{ADC}}_{44.5\mathrm{ms}}\right)}{ {\mathrm{ADC}}_{44.5\mathrm{ms}}}\times 100 \left(\%\right)\end{array}\end{array}$$

where ADC_7.1 ms_ and ADC_44.5 ms_ are the ADC values obtained using the OGSE and PGSE sequences, respectively.

### ROI-based measurement

All images were analyzed using commercially available software (Vitrea; Canon Medical Systems Corporation). The ADC maps were co-registered with the post-contrast T1-weighted images using the rigid body registration. Two independent radiologists (T.H. and Y.K., with 8 and 4 years of radiological experience, respectively), who were blinded to the patients’ clinical and pathological data, performed the ROI analysis. The ROIs were drawn manually on a postcontrast T1-weighted image with the largest tumor diameter, including enhancing region and avoiding necrosis and fluid, and on the corresponding FLAIR image, including non-enhancing peritumoral regions with a FLAIR high signal intensity, and copied them on the corresponding ADC, cADC, and rcADC maps. The mean ADC_44.5ms_ (ADC_44.5ms_^mean^), ADC_7.1ms_ (ADC_7.1ms_^mean^), cADC (cADC^mean^), and rcADC (rcADC^mean^) were calculated for the entire ROI. Furthermore, the fifth and 95^th^ percentile values of ADC_44.5ms_ (ADC_44.5ms_^5th^ and ADC_44.5ms_^95th^), ADC_7.1ms_ (ADC_7.1ms_^5th^ and ADC_7.1ms_^95th^), cADC (cADC^5th^ and cADC^95th^), and rcADC (rcADC^5th^ and rcADC^95th^) were calculated; this method was considered to represent the lowest and highest robust values [26]. The average ROIs size of the enhancing and the peritumoral regions were 587 ± 700 mm^2^ and 870 ± 668 mm^2^, respectively, for PCNSLs, and 651 ± 485 mm^2^ and 611 ± 605 mm^2^, respectively, for GBMs.

### Statistical analysis

The D'Agostino–Pearson normality test was used to check the normality assumption for all parameters in all groups. The Mann–Whitney *U* test was used to compare the mean age between those with PCNSLs and those with GBMs, and the chi-square test was used to determine sex distribution. The intraclass correlation coefficient (ICC) was used to determine the interobserver agreement between the two observers on parametric measurements. Excellent agreement was defined as ICC > 0.74 [27]. The measurements from the two observers were averaged for each case and were used for further analysis. The ADC values were compared among the different diffusion times using the paired-*t* test or Wilcoxon signed-rank test. The mean and the 5^th^ and 95^th^ percentile values of ADC_44.5 ms_, ADC_7.1 ms_, cADC, and rcADC were compared between PCNSLs and GBMs using the unpaired-*t* test or Mann–Whitney *U* test. Receiver operating characteristic (ROC) analysis was performed to determine the optimum threshold for tumor differentiation and to calculate the area under the ROC curve (AUC), sensitivity, specificity, and accuracy for identifying GBM. The best-performing indices were decided for ADC_44.5 ms_, ADC_7.1 ms_, cADC, and rcADC. DeLong’s test was used to compare the AUCs of the best-performing indices. The Bonferroni correction was performed to correct multiple comparisons. A commercially available software package (MedCalc, version 15.10.0; MedCalc statistical software) was used for statistical analysis. Differences with *p*-values of < 0.05 were considered statistically significant.

## Results

**Fig. 3:**
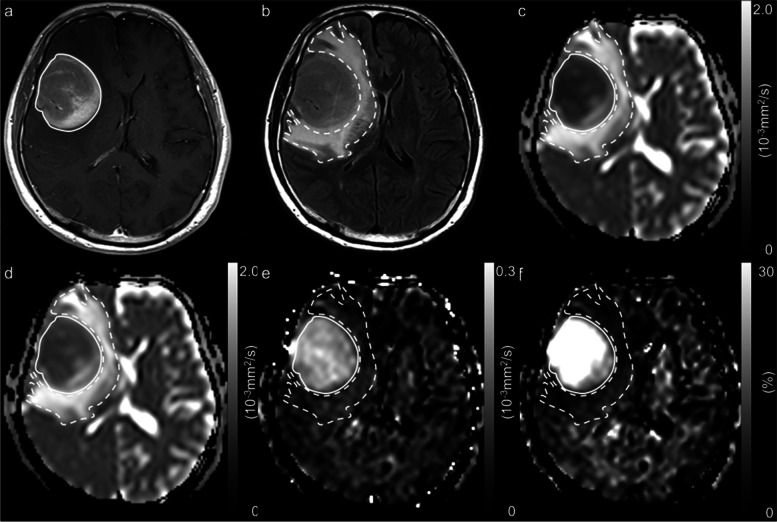
A 67-year-old man with primary central nervous system lymphoma. A contrast-enhanced T1-weighted image with a ROI (solid line) (**a**), a fluid attenuated inversion recovery image with a ROI (dotted line) (**b**), an ADC map derived from pulsed gradient spin-echo (PGSE) DWI at an effective diffusion time (Δ_eff_) of 44.5 ms (**c**), an ADC map derived from oscillating gradient spin-echo (OGSE) DWI at an Δ_eff_ of 7.1 ms (**d**), and maps of the ADC change between PGSE DWI and OGSE DWI (cADC) (**e**) and the relative ADC change between PGSE DWI and OGSE DWI (rcADC) (f). The ADC values in the tumor appear higher at short Δ_eff_ values than at long Δ_eff_ setting. Large changes in the cADC and rcADC are noted between the OGSE and PGSE sequences in the tumor

**Fig. 4:**
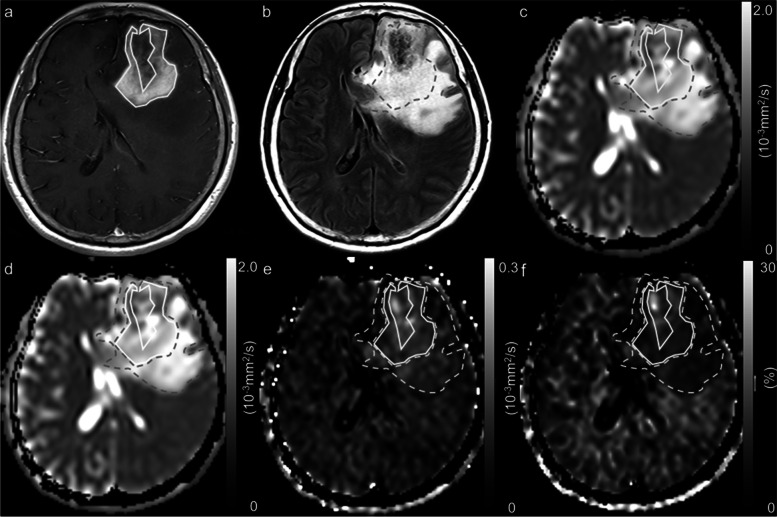
A 65-year-old woman with glioblastoma, isocitrate dehydrogenase-wildtype, grade 4. A contrast-enhanced T1-weighted image with a ROI (solid line) (**a**), a fluid attenuated inversion recovery image with a ROI (dotted line) (**b**), an ADC map derived from pulsed gradient spin-echo (PGSE) DWI at an effective diffusion time (Δ_eff_) of 44.5 ms (**c**), an ADC map derived from oscillating gradient spin-echo (OGSE) DWI at an Δ_eff_ of 7.1 ms (**d**), and maps of the ADC change between PGSE DWI and OGSE DWI (cADC) (**e**) and the relative ADC change between PGSE DWI and OGSE DWI (rcADC) (**f**). The ADC values in the tumor appear higher at short Δ_eff_ values than at long Δ_eff_ setting. Small changes in the cADC and rcADC are noted between the OGSE and PGSE sequences in the tumor

Figures 3 and 4 show the representative diffusion parametric maps for PCNSLs and GBMs.

### Interobserver agreement

The ICCs and 95% confidence intervals for each parameter are shown in Table 2. All parameters showed an excellent agreement.

**Table 2:** The intraclass correlation coefficients and 95% confidence intervals for ADC_44.5ms_^mean^, ADC_44.5ms_^5th^, ADC_44.5ms_^95th^, ADC_7.1ms_^mean^, ADC_7.1ms_^5th^, ADC_7.1ms_^95th^, cADC^mean^, cADC^5th^, cADC^95th^, rcADC^mean^, rcADC^5th^, and rcADC^95th^ of the enhancing and peritumoral regions

Parameters	Intraclass correlation coefficients (95% confidence intervals)	
	Enhancing region	Peritumoral region
ADC_44.5ms_^mean^	0.962 (0.943–0.975)	0.994 (0.991–0.996)
ADC_44.5ms_^5th^	0.930 (0.895–0.954)	0.966 (0.950–0.978)
ADC_44.5ms_^95th^	0.870 (0.809–0.912)	0.975 (0.962–0.983)
ADC_7.1ms_^mean^	0.964 (0.945–0.976)	0.993 (0.989–0.995)
ADC_7.1ms_^5th^	0.933 (0.899–0.956)	0.963 (0.944–0.975)
ADC_7.1ms_^95th^	0.894 (0.843–0.924)	0.982 (0.973–0.988)
cADC^mean^	0.958 (0.934–0.972)	0.981 (0.971–0.987)
cADC^5th^	0.860 (0.795–0.906)	0.979 (0.968–0.986)
cADC^95th^	0.955 (0.931–0.970)	0.983 (0.975–0.989)
rcADC^mean^	0.970 (0.954–0.980)	0.993 (0.989–0.995)
rcADC^5th^	0.888 (0.834–0.925)	0.979 (0.969–0.986)
rcADC^95th^	0.907 (0.870–0.934)	0.980 (0.970–0.987)

### Comparisons of the diffusion parameters

**Fig. 5:**
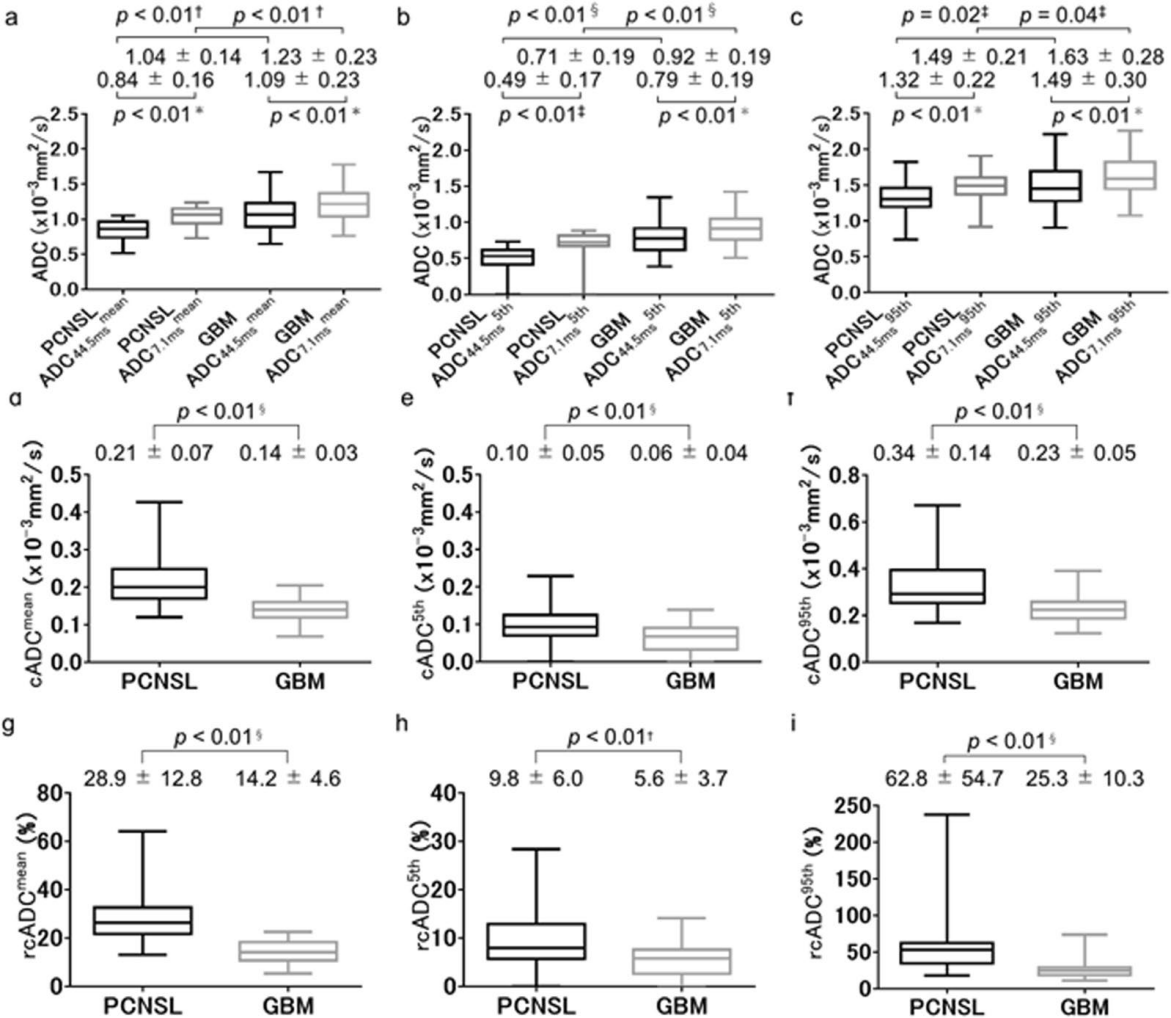
Box-whisker plots of the ADC_44.5ms_^mean^ and ADC_7.1ms_^mean^ (**a**), ADC_44.5ms_^5th^ and ADC_7.1ms_^5th^ (**b**), and ADC_44.5ms_^95th^ and ADC_7.1ms_^95th^ (**c**) in enhancing regions for primary central nervous system lymphomas (PCNSLs) and glioblastomas (GBMs). For each tumor, each index for ADC_7.1 ms_ was significantly higher than the corresponding index for ADC_44.5 ms_ (each *p* < 0.01, respectively) (**a-c**). Comparisons of the cADC^mean^ (**d**), cADC^5th^ ©, and cADC^95th^ (**f**) between PCNSLs and GBMs. Each index for the cADC was significantly higher in PCNSLs than in GBMs (each *p* < 0.01, respectively). Comparisons of the rcADC^mean^ (**g**), rcADC^5th^ (**h**), and rcADC^95th^ (**i**) for PCNSLs and GBMs. Each index for the rcADC was significantly higher in PCNSLs than in GBMs (each *p* < 0.01, respectively). Statistical tests used: ^*^paired *t*-test, ^†^unpaird-*t* test, ^‡^Wilcoxon signed-rank test, ^§^Mann–Whitney *U* test

**Fig. 6:**
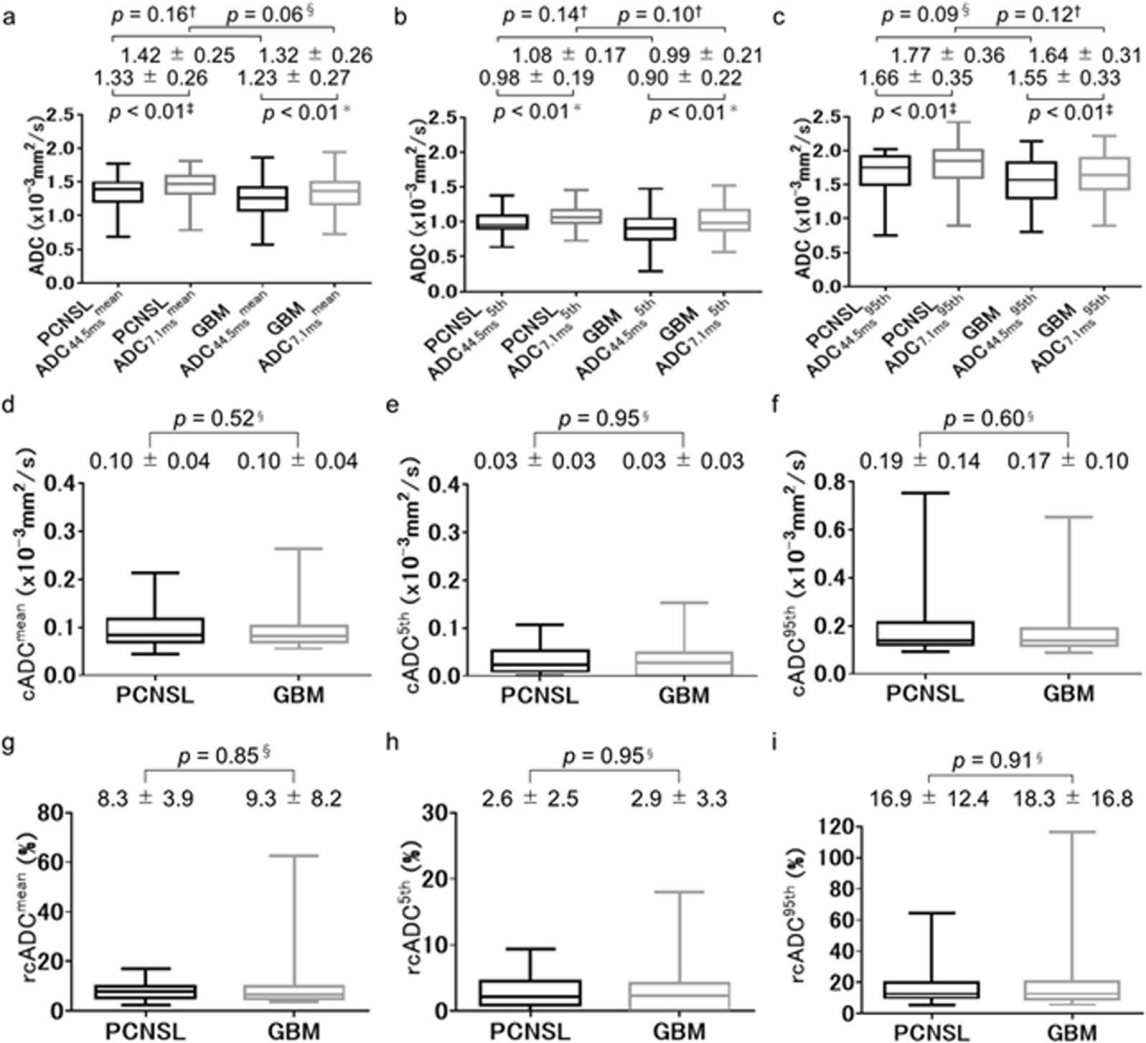
Box-whisker plots of the ADC_44.5ms_^mean^ and ADC_7.1ms_^mean^ (**a**), ADC_44.5ms_^5th^ and ADC_7.1ms_^5th^ (**b**), and ADC_44.5ms_^95th^ and ADC_7.1ms_^95th^ (**c**) in peritumoral regions for primary central nervous system lymphomas (PCNSLs) and glioblastomas (GBMs). All three indices for ADC_7.1 ms_ were significantly higher than those for ADC_44.5 ms_ for both tumors (all *p* < 0.01, respectively) (**a**–**c**). No significant difference in any of the three indices of ADC_44.5 ms_, ADC_7.1 ms_, cADC, and rcADC was observed between PCNSLs and GBMs (**a**–**i**). Statistical tests used: ^*^paired *t*-test, ^†^unpaird-*t* test, ^‡^Wilcoxon signed-rank test, ^§^Mann–Whitney *U* test

In enhancing regions, all three indices for ADC_7.1 ms_ were significantly higher than those for ADC_44.5 ms_ for both tumors (all *p* < 0.01) (Fig. 5a–c). All three indices for ADC_7.1 ms_ and ADC_44.5 ms_ were significantly lower for PCNSLs than for GBMs (all *p* < 0.05) (Fig. 5a–c). Furthermore, all three indices for cADC and rcADC values were significantly higher for PCNSLs than for GBMs (all *p* < 0.01) (Fig. 5d–i). In peritumoral regions, all three indices for ADC_7.1 ms_ were significantly higher than those for ADC_44.5 ms_ for both tumors (all *p* < 0.01) (Fig. 6a–c). No significant difference was observed in any of the three indices for ADC_44.5 ms_, ADC_7.1 ms_, cADC, and rcADC between PCNSLs and GBMs (Fig. 6a–i).

### Diagnostic performance in differentiating PCNSLs from GBMs

**Fig. 7:**
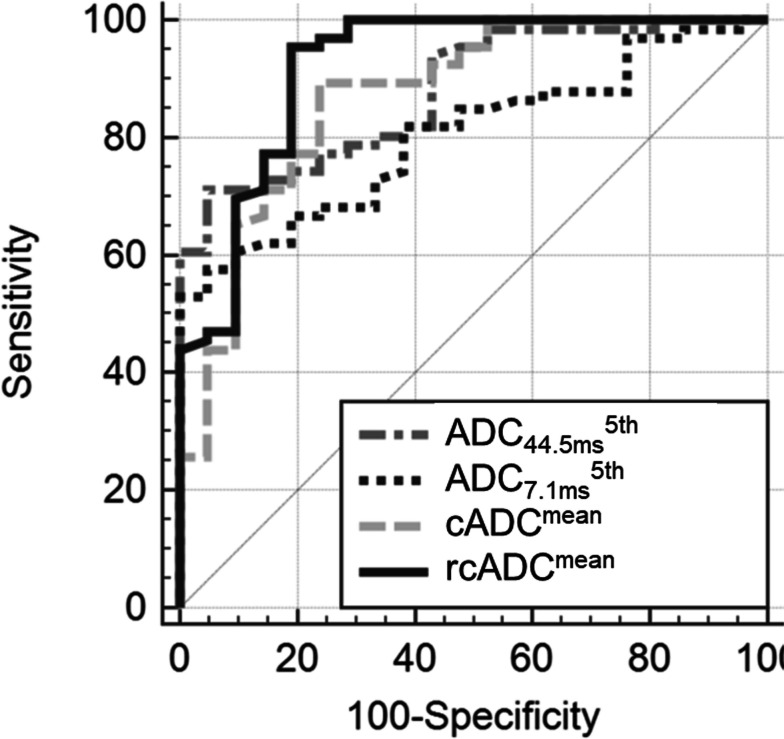
Receiver operating characteristic curves for the best-preforming indices for the ADC_44.5ms_^5th^, ADC_7.1ms_^5th^, cADC^mean^, and rcADC^mean^ in enhancing regions

Table 3 shows the results of the ROC curve analysis. In enhancing region, the ADC_44.5ms_^5th^, ADC_7.1ms_^5th^, cADC^mean^, and rcADC^mean^ values were the best-performing indices for the ADC_44.5 ms_, ADC_7.1 ms_, cADC, and rcADC, respectively. Figure 7 shows the ROC curves for the ADC_44.5ms_^5th^, ADC_7.1ms_^5th^, cADC^mean^, and rcADC^mean^ values. Pairwise comparisons of the AUCs among the best-performing indices revealed that the AUC of the ADC_44.5ms_^5th^ was significantly greater than that of the ADC_7.1 ms_^5th^ (*p* < 0.001), whereas no other comparisons of the AUCs revealed significant differences (Table 4). The rcADC^mean^ showed the highest performance (AUC: 0.920).

**Table 3:** The AUC, optimal threshold, sensitivity, specificity, and accuracy for the ADC_44.5ms_^mean^, ADC_44.5ms_^5th^, ADC_44.5ms_^95th^, ADC_7.1ms_^mean^, ADC_7.1ms_^5th^, ADC_7.1ms_^95th^, cADC^mean^, cADC^5th^, cADC^95th^, rcADC^mean^, rcADC^5th^, and rcADC^95th^ of the enhancing and peritumoral regions to differentiate primary central nervous system lymphomas from glioblastomas

Parameter	AUC (95% CI)	*p*-value	Threshold value	Sensitivity (%)	Specificity (%)	Accuracy (%)
Enhancing region						
ADC_44.5ms_^mean^	0.811 (0.713–0.887)	< 0.01	1.052 (× 10^–3^ mm^2^/s)	53.0	100	64.4
ADC_44.5ms_^5th^	0.881 (0.793–0.940)	< 0.01	0.646 (× 10^–3^ mm^2^/s)	71.2	95.2	77.0
ADC_44.5ms_^95th^	0.674 (0.565–0.771)	< 0.01	1.325 (× 10^–3^ mm^2^/s)	72.7	61.9	70.1
ADC_7.1ms_^mean^	0.747 (0.643–0.835)	< 0.01	1.238 (× 10^–3^ mm^2^/s)	45.5	100	58.6
ADC_7.1ms_^5th^	0.803 (0.704–0.880)	< 0.01	0.888 (× 10^–3^ mm^2^/s)	53.0	100	64.4
ADC_7.1ms_^95th^	0.646 (0.537–0.746)	0.02	1.636 (× 10^–3^ mm^2^/s)	45.5	90.5	56.3
cADC^mean^	0.871 (0.782–0.933)	< 0.01	0.174 (× 10^–3^ mm^2^/s)	89.4	76.2	86.2
cADC^5th^	0.725 (0.619–0.815)	< 0.01	0.081 (× 10^–3^ mm^2^/s)	68.2	71.4	69.0
cADC^95th^	0.804 (0.705–0.881)	< 0.01	0.263 (× 10^–3^ mm^2^/s)	80.3	76.2	79.3
rcADC^mean^	0.920 (0.842–0.967)	< 0.01	21.2 (%)	95.5	81.0	92.0
rcADC^5th^	0.725 (0.619–0.816)	< 0.01	6.85 (%)	66.7	71.4	67.8
rcADC^95th^	0.884 (0.797–0.943)	< 0.01	32.0 (%)	84.8	85.7	85.1
Peritumoral region						
ADC_44.5ms_^mean^	0.613 (0.503–0.716)	0.11	1.347 (× 10^–3^ mm^2^/s)	65.2	61.9	64.4
ADC_44.5ms_^5th^	0.615 (0.505–0.718)	0.08	0.907 (× 10^–3^ mm^2^/s)	54.5	76.2	59.8
ADC_44.5ms_^95th^	0.622 (0.512–0.724)	0.10	1.596 (× 10^–3^ mm^2^/s)	56.1	71.4	59.8
ADC_7.1ms_^mean^	0.635 (0.525–0.736)	0.06	1.445 (× 10^–3^ mm^2^/s)	66.7	66.7	66.7
ADC_7.1ms_^5th^	0.630 (0.520–0.731)	0.05	0.969 (× 10^–3^ mm^2^/s)	47.0	85.7	56.3
ADC_7.1ms_^95th^	0.633 (0.523–0.734)	0.08	1.684 (× 10^–3^ mm^2^/s)	56.1	71.4	59.8
cADC^mean^	0.547 (0.437–0.654)	0.55	0.082 (× 10^–3^ mm^2^/s)	53.0	66.7	56.3
cADC^5th^	0.504 (0.395–0.613)	0.95	0.007 (× 10^–3^ mm^2^/s)	30.3	81.0	42.5
cADC^95th^	0.539 (0.428–0.646)	0.60	0.128 (× 10^–3^ mm^2^/s)	43.9	71.4	50.6
rcADC^mean^	0.514 (0.405–0.623)	0.85	7.35 (%)	59.1	57.1	58.6
rcADC^5th^	0.504 (0.395–0.613)	0.95	0.75 (%)	66.7	19.0	55.2
rcADC^95th^	0.509 (0.399–0.618)	0.90	27.6 (%)	81.8	4.8	63.2

**Table 4:** Pairwise comparison of the AUCs among ADC_44.5ms_^5th^, ADC_7.1ms_^5th^, cADC^mean^, and rcADC^mean^ of the enhancing regions

Parameter	rcADC^mean^	cADC^mean^	ADC_7.1ms_^5th^
ADC_44.5ms_^5th^			
DBE	0.034	0.009	0.078
*p*	0.243	0.845	< 0.001
ADC_7.1ms_^5th^			
DBE	0.117	0.069	
*p*	0.019	0.277	
cADC^mean^			
DBE	0.049		
*p*	0.043		

*DBE* Difference between areas

## Discussion

This study showed that the ADC values with both long (44.5 ms) and short (7.1 ms) effective diffusion times (ADC_44.5 ms_ and ADC_7.1 ms_, respectively) were significantly lower in PCNSLs than in GBMs, and both the cADC and rcADC were significantly higher in PCNSLs than in GBMs. The rcADC, particularly the rcADC^mean^, showed the highest differentiation performance among all indices, suggesting the clinical usefulness of time-dependent diffusion MRI for this purpose.

The clinical value of ADC in diagnosing PCNSL has been well documented. A distinctly low ADC in the enhancing region of PCNSL reflecting higher cellularity helps differentiate PCNSLs from other brain tumors, including GBMs and metastatic brain tumors [28]. We confirmed that ADCs measured within the enhancing region using the PGSE DWI sequence with an effective diffusion time of 44.5 ms, which is within the range of typical diffusion time of clinical DWI, is useful in distinguishing PCNSLs from GBMs. Notably, our results showed that the diagnostic performance of the ADC_7.1 ms_ obtained using the OGSE DWI sequence was significantly lower than that of the ADC_44.5 ms_ obtained using the conventional PGSE DWI sequence. This finding clearly demonstrates the importance of effective diffusion time setting in clinical DWI. It is presumed that the ADC obtained with shorter effective diffusion times is less sensitive to the microstructural differences between GBMs and PCNSLs.

Previous studies have reported inconsistent results regarding the comparison of ADC in the non-enhancing peritumoral regions between PCNSLs and GBMs. Studies by Ko et al. and Cindil et al. showed that ADC in the peritumoral regions was significantly higher in PCSNLs than in GBMs [29, 30], whereas Wang et al. reported that it did not significantly differ between the two tumor types [31]. The present study failed to show significant difference in any of the peritumoral ADC indices including those of ADC diffusion time dependence between PCNSLs and GBMs. Our results suggest that ADC diffusion time dependence in the peritumoral regions may not be useful for differentiating between PCNSLs and GBMs.

Researchers have reported the usage of time-dependent diffusion MRI in evaluating intracranial tumors. Maekawa et al. showed that both the ADC change and the relative ADC change in ADC between short (6.5 ms) and long (32.5 ms) effective diffusion times were significantly higher in high-grade intra-axial brain tumors than low-grade ones [25]. The same group examined extra-axial tumors using time-dependent diffusion MRI and reported that the relative percentage changes between short (6.5 ms) and long (32.5 ms) effective diffusion times of diffusion tensor eigenvalues (λ1, λ2, and λ3) and the mean diffusivity were significantly higher in pituitary adenomas than in meningothelial meningiomas and acoustic neuromas [32]. More recently, Zhang et al. examined pediatric gliomas using diffusion-time-dependent diffusion MRI and developed a two-compartment microstructural model to obtain intracellular fraction, cell diameter, and cellularity [33]. They demonstrated that the cellularity index achieved the highest performance in identifying the histological grade, and cell diameter achieved the highest discriminating performance for the molecular classification of H3K27-altered gliomas in midline gliomas. Zhu et al. examined five patients with glioma using an ultra-high-performance gradient MRI system, and demonstrated that the ratio of ADC measured at short diffusion times to that measured at long diffusion times is promising for revealing the heterogenous tumor microstructures including cellular density in presurgical and post-treatment gliomas [34]. These studies have suggested the clinical possibility and validity of time-dependent diffusion MRI in characterizing intracranial tumors. However, no studies have investigated the usage of time-dependent diffusion MRI in differentiating PCNSLs from GBMs.

Compared with ADC itself, the diffusion time dependence of ADC is considered more specific to restricted diffusion caused by microscopic barriers in biological tissues, such as cell membranes. Researchers have attributed the stronger diffusion time dependence of ADC in high-grade tumors than in low-grade ones to more abundant diffusion-restricting microstructures within the range of diffusion lengths determined by the selected diffusion times in the OGSE and PGSE DWI sequences [24, 25]. PCNSLs are characterized by monotonous high cellularity and small extracellular space, whereas GBMs are characterized by heterogeneous moderate cellularity and medium-sized extracellular space due to the extracellular matrix, fine hemorrhage, and necrosis [35, 36]. Thus, PCNSLs may have a narrower extracellular space than GBMs, where molecular diffusion is less restricted than that in the intracellular space. The higher cellularity and narrower extracellular space of PCNSLs may account for the stronger diffusion time dependence of ADC. Other reasons for the stronger ADC diffusion time dependence in PCNSLs may include the difference in cell size between PCNSLs and GBMs. Studies have shown that the cell sizes were 10–20 μm for PCNSLs and 10–33 μm for GBMs [37, 38]. The cADC at a given set of diffusion times critically depends on the spacing between the barriers and thus could vary with cell size [39]. At our effective diffusion time settings, the smaller cell size of PCNSLs than GBMs might have resulted in stronger ADC diffusion time dependence. Further studies are needed to discover the pathological basis for our findings.

### Limitations of the study

This study had several limitations. First, the sample size was relatively small. Studies with larger sample sizes are required to confirm our findings. Second, only two effective diffusion times (7.1 ms and 44.5 ms) and a fixed set of b-values (0 and 1,500 s/mm^2^) were investigated. The use of shorter or longer effective diffusion times may have changed the results. However, the gradient performance of our clinical MRI system limits the range of the effective diffusion time in OGSE. Finally, although all tumors were pathologically diagnosed, detailed comparisons between the tissue microstructures and imaging findings were not performed.

## Conclusions

The mean of relative changes in the ADC value between short and long diffusion times achieved better performance than ADC from conventional PGSE DWI in differentiating between PCNSLs and GBMs. Our study demonstrated the usefulness of time-dependent diffusion MRI using OGSE in differentiating the two tumor types.

## Data Availability

The datasets of current study are available from the corresponding author on reasonable request.

## Abbreviations

ADC: Apparent diffusion coefficient
ADC_7.1ms_: Apparent diffusion coefficient values obtained with an effective diffusion time of 7.1 ms
ADC_44.5ms_: Apparent diffusion coefficient values obtained with an effective diffusion time of 44.5 ms
cADC: Apparent diffusion coefficient change
rcADC: Relative apparent diffusion coefficient change
AUC: Area under the receiver operating characteristic curve
DWI: Diffusion weighted imaging
FLAIR: Fluid attenuated inversion recovery
FOV: Field of view
GBM: Glioblastoma
ICC: Intraclass correlation coefficient
MRI: Magnetic resonance imaging
OGSE: Oscillating gradient spin-echo
PGSE: Pulsed gradient spin-echo
PCNSL: Primary central nervous system lymphoma
ROC: Receiver operating characteristic
ROI: Region of interest
TE: Echo time
TR: Repetition time
δ: Diffusion gradient pulse duration
Δ: Diffusion gradient separation
Δ_eff_: Effective diffusion time
